# MiR-137 Deficiency Causes Anxiety-Like Behaviors in Mice

**DOI:** 10.3389/fnmol.2019.00260

**Published:** 2019-10-30

**Authors:** Hai-Liang Yan, Xiao-Wen Sun, Zhi-Meng Wang, Pei-Pei Liu, Ting-Wei Mi, Cong Liu, Ying-Ying Wang, Xuan-Cheng He, Hong-Zhen Du, Chang-Mei Liu, Zhao-Qian Teng

**Affiliations:** ^1^State Key Laboratory of Stem Cell and Reproductive Biology, Institute of Zoology, Chinese Academy of Sciences, Beijing, China; ^2^Savaid Medical School, University of Chinese Academy of Sciences, Beijing, China; ^3^Institute of Stem Cell and Regenerative Medicine, Chinese Academy of Sciences, Beijing, China

**Keywords:** miR-137, EZH2, synaptic transmission, synaptic plasticity, anxiety and depression

## Abstract

Anxiety and depression are major public health concerns worldwide. Although genome-wide association studies have identified several genes robustly associated with susceptibility for these disorders, the molecular and cellular mechanisms associated with anxiety and depression is largely unknown. Reduction of microRNA-137 (miR-137) level has been implicated in the etiology of major depressive disorder. However, little is known about the *in vivo* impact of the loss of miR-137 on the biology of anxiety and depression. Here, we generated a forebrain-specific miR-137 knockout mouse line, and showed that miR-137 is critical for dendritic and synaptic growth in the forebrain. Mice with miR-137 loss-of-function exhibit anxiety-like behavior, and impaired spatial learning and memory. We then observe an elevated expression of EZH2 in the forebrain of miR-137 knockout mice, and provide direct evidence that knockdown of EZH2 can rescue anxious phenotypes associated with the loss of miR-137. Together our results suggest that loss of miR-137 contributes to the etiology of anxiety, and EZH2 might be a potential therapeutic target for anxiety and depressive phenotypes associated with the dysfunction of miR-137.

## Introduction

Anxiety and depression are common mood disorders that affect millions of people irrespective of age, race, ethnicity and gender, resulting in the inability to concentrate, insomnia, feelings of extreme sadness and guilt, helplessness and hopelessness, and suicide attempt (WHO, 2012; Jesulola et al., 2018). Despite continued research in neurophysiology and neuropsychiatry increasing the understanding of the pathophysiology of anxiety and depression, mechanisms associated with the pathogenesis of anxiety and depression have yet to be completely understood and current treatments remain ineffective in a large subset of patients (Brigitta, 2002; Menard et al., 2016; Jesulola et al., 2018).

Genome-wide association studies have suggested that the microRNA miR-137 is closely associated with the etiology of several neuropsychiatric disorders, such as schizophrenia (Ripke et al., 2013), autism spectrum disorder (Pinto et al., 2014), and bipolar disorder (Duan et al., 2014). MiR-137 has important regulatory roles in brain function, dysfunction of miR-137 also contributes to brain cancers, including neuroblastoma and glioblastoma multiforme (Silber et al., 2008; Althoff et al., 2013; Zhang et al., 2017).

Either overexpression or downregulation of miR-137 impairs brain function, suggesting miR-137-mediated regulation during neurodevelopment is dosage-dependent (Cheng et al., 2018). Overexpression of miR-137 promotes the proliferation of adult neural stem cells (aNSC), whereas a reduction of miR-137 enhances aNSC differentiation (Szulwach et al., 2010). MiR-137 overexpression has been suggested to be associated with the etiology of schizophrenia (Guan et al., 2014). Overexpression of miR-137 inhibits dendritic morphogenesis, phenotypic maturation, and spine development (Smrt et al., 2010). MiR-137 overexpression also reduces evoked synaptic transmission and spontaneous release by regulating synaptogenesis, synaptic ultrastructure and synapse function (He et al., 2018). On the other hand, we have previously found that partial loss of miR-137 in mice causes repetitive behavior and impairs sociability and learning *via* increased PDE10A, a cyclic nucleotide phosphodiesterase that is highly expressed in the brain (Cheng et al., 2018). Loss of miR-137 in the brain leads to synaptic and dendritic overgrowth (Cheng et al., 2018).

Alterations of miR-137 and its target gene levels have been implicated in the etiology of major depressive disorder. Smalheiser et al. (2012) reported that miR-137 levels are substantially down-regulated by 25% in the postmortem prefrontal cortex (PFC) of depressed patients with suicidal behavior. Several reports also provided strong association between CACNA1C, a potential target gene of miR-137, with the risk of major depression (Casamassima et al., 2010; Green et al., 2010; Shi et al., 2011). In addition, Zhao et al. (2013) found that miR-137 levels were significantly lower in the brain in post-stroke depression rats, and exogenous delivery of miR-137 could improve their behavioral performance by suppressing the expression of Grin2A. However, little is known about the *in vivo* impact of the loss of miR-137 on the biology of mental dysfunction.

In this study, we generated a forebrain-specific miR-137 knockout mouse line to investigate the impact of miR-137 loss of function *in vivo*. We show that miR-137 is critical for dendritic and synaptic growth in the forebrain. MiR-137 loss-of-function results in altered synaptic transmission and plasticity, and anxiety-like behavior in mice. One of the miR-137 mRNA targets, *Ezh2*, is significantly upregulated in the forebrain of miR-137 knockout mice. Knockdown of EZH2 can rescue the deficits associated with the loss of miR-137. Considering altered expression of *Ezh2* and miR-137 in patients with mood disorders (Zhao et al., 2013; Murphy et al., 2015), our results suggest that the dysregulation of miR-137-*Ezh2* axis might contribute to mood disorders in humans.

## Materials and Methods

### Animals

All mice used were on the 129S6/SvEvTac genetic background. We previously generated a mouse model which has two loxP sites inserted upstream (~2 kb) and downstream (~0.6 kb) of the *Mir137* gene (Cheng et al., 2018). *miR-137*^flox/flox^;*Emx1-Cre* mice were generated to specifically delete *Mir137* in the forebrain by crossing *miR-137*^flox/flox^ mice with *Emx1-Cre* mice (Jax Stock No. 005628). Genotyping was performed using tail DNA, and the primers were designed as followings: (Cre: forward 5′-GCGGTCTGGCAGTAAAAACTATC-3′, reverse 5′-GTGAAACAGCATTGCTGTCACTT-3′; Emx1: forward, 5′-AAGGTGTGGTTCCAGAATCG-3′, reverse 5′-CTCTCCACCAGAAGGCTGAG-3′; *miR-137 loxP*: forward 5′-GCTGTGTGGGCATTTATGCCATG-3′, reverse 5′-AAGGCAATAGTCTATGAGCAACGTG-3′). All the procedures involving mice were in accordance with the protocol of the Institute of Zoology, Chinese Academy of Sciences.

### Primary Neuron Culture

Primary neuronal culture was performed as described previously (Liu et al., 2017). Briefly, hippocampi were isolated from P0 newborn *miR137*^flox/flox^ or *miR-137*^flox/flox^;*Emx1-cre* mice and dissociated with trituration after trypsin/EDTA treatment. Then, the cells were plated onto poly-D-lysine coated glass coverslips with a density of 5 × 10^4^ cells per well in a 24-well plate. Neurons were cultured in neurobasal (Invitrogen) medium supplemented with 1% B27, 1% GlutaMax (Invitrogen) and 1% penicillin/streptomycin.

### Lentiviral Construct

*Ezh2* shRNA sequence (GCAAATTCTCGGTGTCAAACA) was inserted in the U6-shRNA lentiviral construct. Lentiviruses were produced by PEI-mediated co-transfection of HEK293T cells with pREV, pVSVG, pMDL, and lentiviral plasmids. The medium containing virus was collected at 48 h and 72 h post-transfection, and then filtered through a 0.22 μm cellulose acetate filters (Millipore), and then concentrated in PBS after 2 h ultracentrifugation at 20,000 rpm.

### Western Blot

Brain tissues were lysed with RIPA buffer (P0013B, Beyotime). Protein samples were separated in 8%–12% SDS-PAGE gels and transferred to polyvinylidene fluoride (PVDF) membranes (Millipore). The membranes were then blocked in 3% milk in TBS-T and incubated with primary EZH2 antibodies (Cell signaling, #5246s) at 4°C overnight. The secondary antibody was horseradish peroxidase (HRP)-conjugated goat anti-mouse. The immunoreactive products were detected with enhanced chemiluminescence reagent (ECL, pierce). The band intensity of the blots was quantified by the software ImageJ. β-actin was used as an internal control.

### RNA Isolation and qRT-PCR

Total RNA was isolated with TRIzol reagent (Invitrogen) according to the manufacturer’s instruction. cDNA was obtained from reverse transcription of 2 μg total RNA using a Transcriptor First Strand cDNA Synthesis Kit (TransGen Biotech). For real-time PCR analysis, cDNA was quantified by qPCR *via* SYBR Green assay. Quantification of qPCR data was analyzed following the ΔΔCt method using GAPDH or U6 as normalization control. Based on the sequence of transcripts and the primer bank, the following primers are used: *Ezh2* (forward: 5′-GCCAGACTGGGAAGAAATCTG-3′; reverse: 5′-TGTGCTGGAAAATCCAAG-3′), GAPDH (forward: 5′-AAGGTCATCCCAGAGCTGAA-3′; reverse: 5′-AGGAGACAACCTGGTCCTCA-3′), U6 (forward: 5′-CTCGCTTCGGCAGCACA-3′; reverse: 5′-AACGCTTCACGAATTTGCGT-3′), miR-137 (forward: 5′-CGGGCTTATTGCTTAAGAATA-3′; reverse: 5′-GCAGGGTCCGAGGTATTC-3′).

### Neuron Morphology Analysis

We analyzed *in vivo* dendrites by three-dimensional (3D) reconstructions of representative GFP positive neurons from the dentate gyrus (DG) of the hippocampus. Only neurons that had no truncated branches were randomly selected for 3D reconstructions. One-hundred and fifty micrometer-thick brain sections across hippocampus were prepared for neuronal morphology analysis, and 30 μm-thick for spine density analysis. Images of the dendrites and spines were acquired using on Zeiss LSM710 confocal microscope with 20× and 100× oil lens, respectively. Dendrites were traced and analyzed using the software ImageJ (NIH) with the Simple Neurite Tracer Plugin.

For dendritic analysis *in vitro*, cultured neurons were fixed with 4% PFA at Day 7, washed with cold PBS, and blocked with 2% BSA containing 0.05% Triton X-100 for 1 h at room temperature. Followed by overnight incubation with primary antibodies (MAP2, Mab3418, Millipore), after washing with PBS, cells were incubated with secondary antibodies, goat anti-mouse Alexa Fluor 568 (Invitrogen). Images of dendrites were acquired on LSM710 confocal microscope with 20× lens. For spine analysis, cultured neurons were fixed with 4% PFA at DIV-14. Images of spines were acquired on LSM710 confocal microscope with 63× oil lens.

### Electrophysiology

Acute hippocampal slices were prepared from 3- to 5-month-old miR-137 cKO and their WT littermates. In brief, mice were anesthetized and decapitated; the brain was quickly transferred into ice-cold oxygenated (95% O_2_, 5% CO_2_) Na^+^ free sucrose solution containing the following (in mM): 250 sucrose, 26 NaHCO_3_, 0.5 CaCl_2_, 2.5 KCl, 1.25 NaH_2_PO4, and 4.0 MgCl_2_. 400 μm-thick transverse sections across hippocampi were sliced with a vibratome (Campden instruments, 7000smz) in ice bath. Slices were equilibrated in oxygenated ACSF (125 mM NaCl, 2.5 mM KCl, 1 mM MgCl_2_, 2 mM CaCl_2_, 25 mM NaHCO_3_, 1.25 mM NaH_2_PO_4_, and 25 mM glucose, pH 7.4) for at least 1 h before recording at RT (~25°C). Extracellular field EPSPs (fEPSPs) were recorded in a submerged chamber, with a glass micropipette (resistance: 3–5 MΩ) filled with ACSF placed at the stratum radiatum of CA1. A concentric bipolar tungsten electrode was used for stimulation of Schaffer collaterals. During the whole recording process, stimulus intensity were kept constant and set to generate 50% of the maximum fEPSP. After 15 min of a steady baseline was recorded, long-term potentiation (LTP) was induced by applying a train of high-frequency stimulation (HFS; 100 Hz for 1 s). Mutliclamp 700B amplifiers and Digidata 1440A converter were used for data acquisition. Data were sampled at 10 kHz, filtered at 2 kHz, and analyzed with Clampfit10.6. The slope of fEPSPs was measured to quantify the strength of synaptic transmission, and the average LTP was determined at 55– 60 min after induction, relative to the 5 min immediately before LTP induction.

### Stereotactic Injection

Six- to eight-week week-old *miR137*^flox/+^ and *miR-137*^flox/+^;*Emx1-cre* male mice were anesthetized with 200 mg/kg Avertin (Wako Pure Chemical Industries, Limited Osaka, Japan). One microliter concentrated lentivirus with titer of ~1 × 10^9^/μl was injected stereotaxically into the hippocampus with the following coordinates (stereotaxic coordinates from Bregma: 2.0 mm caudal, 1.2 mm lateral, 2.0 mm ventral; 2.8 mm caudal, 2.0 mm lateral, 1.7 mm ventral). One month after viral grafting, mice were subjected to behavioral tests.

### Behavioral Tests

Animals were kept in groups of 3–5 animals on a 12:12h light:dark cycle. All behaviors tests were done during the light phase, and only male mice at 2- to 3- month-old were used for behavioral assays. Videos were recorded and analyzed by the software Smart V3.0.03 (Panlab, Barcelona, Spain).

The light-dark preference test was performed similar to that previously described (Tang et al., 2017). In brief, an apparatus (45 × 27 × 27 cm) consisting of two chambers, a dark compartment (18 × 27 cm) with black walls and a light compartment (27 × 27 cm) with white walls. The two chambers are connected through an open door (7 × 7 cm) situated on the floor level at the center of the separating wall. Mice were gently placed in the dark box and allowed to freely move around for 10 min. Transitions between the two chambers and the time spent in each box were recorded.

The open field test was conducted in a 72 × 72 × 36 cm box. A test subject was placed in the center of the box, and its behavior was video-recorded for 10 min by a camera positioned directly above the arena. The duration of time that the test mouse spent in the center zone (18 × 18 cm) of the box was recorded as a measure of distress (less time in the center = greater distress), and the number of entries into the center zone of the arena was counted as a measure of exploratory behavior (greater frequency of entries = greater exploratory behavior).

In Morris water maze test, a circular water tank (diameter 120 cm, height 50 cm) was filled with opaque water (21 ± 1°C; 25 cm of depth), which was made with nontoxic white paint. A13 cm-in-diameter platform was hidden 1 cm beneath the surface of the water at the middle of a given quadrant of the water tank. Mice were trained in finding the escape platform in four trials per day for five consecutive days. For each trial, the mouse was placed into a randomly chosen quadrant and allowed to swim for up to 1 min to find and stand on the platform. If it failed to find the platform within that time, escape was assisted. Mice were guided to the platform and kept resting for 15 s. During the probe test, the platform was removed and mice were allowed to explore the maze for 1 min. Swimming speed, first latency to the platform, time spent and entries in the platform quadrant were recorded.

### Statistical Analysis

Statistical analyses were analyzed using GraphPad Prism (V7.04, GraphPad Software, San Diego, CA, USA). Statistical significance was defined as *p < 0.05, **p < 0.01, and ***p < 0.001 by either unpaired student’s two-tailed *t*-test or AVONA measures with multiple comparison *post hoc* test. All data were presented as mean ± SEM.

## Results

### Loss of miR-137 in the Nervous System Leads to the Anxiety-Like Behavior in Mice

**Figure 1:**
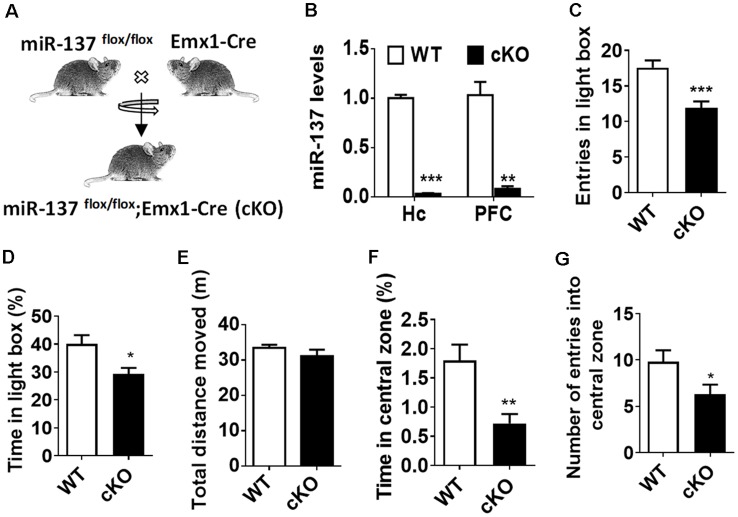
Loss of microRNA-137 (miR-137) in the nervous system leads to the anxiety-like behavior in mice. **(A)** Generation of the miR-137 conditional knockout mice. By crossing the miR-137 floxed mice with Emx1-Cre mice, we specifically deleted miR-137 in the forebrain and generated miR-137 cKO mice. **(B)** Assessment of mature miR-137 levels by quantitative RT-PCR (qPCR) indicated almost undetectable or very low levels in both hippocampus (Quinn et al., 2018) and prefrontal cortex (PFC) of miR-137 cKO mice (*n* = 4 mice per group; **p < 0.01, ***p < 0.001; student’s *t*-test). **(C)** Number of entries and **(D)** percentage of time spent in the light compartment in the light-dark preference test were significantly decreased in miR-137 cKO mice (*n* = 12–14 mice per group; *p < 0.05, ***p < 0.001; student’s *t*-test). **(E)** In the open field test, there was no difference in the total distance moved during the 30-min period, suggesting similar locomotor activities between mir-137 cKO and WT mice. However, mir-137 cKO mice spent less time in center **(F)**, and entered the center zone significantly fewer times than WT mice **(G)**, indicating elevated anxious behavior (*n* = 12–14 mice per group; *p < 0.05, **p < 0.01; student’s *t*-test).

By crossing miR-137^flox/flox^ mice with Emx1-Cre line, we generated the forebrain-specific knockout (cKO) mice (*miR-137^flox/flox^;Emx1-Cre*, miR-137 cKO; Figure 1A). Using quantitative RT-PCR (qPCR), mature miR-137 expression was almost undetectable in the hippocampus and PFC of miR-137 cKO mice compared to control littermates (Figure 1B). MiR-137-deficient mice and WT mice did not significantly differ in overall appearance, body weight, brain weight, or survival rate of miR-137 cKO mice (data not shown).

To examine any behavioral phenotypes of miR-137 cKO mice, we subjected adult males to a battery of behavioral tests. During the training phase of the Morris water maze test (Supplementary Figure S1), four trials were performed per day for four successive days, miR-137 cKO mice showed significantly higher latency to the platform (Supplementary Figure S1A). The subsequent probe test demonstrated that miR-137 cKO mice exhibited a significant delay to targets and fewer target crossings (Supplementary Figures 1B,C), indicating impaired learning and memory. We then measured mental-related behaviors of miR-137 cKO mice. On the light-dark box test (LDB), miR-137 cKO mice displayed a significant decrease in both the number of entries and the time spent in the light chamber (Figures 1C,D). On the open-field test (OFT), there was no difference in total distance moved between the miR-137 cKO mice and their WT littermates (Figure 1E) indicating that loss of miR-137 did not differentially affect locomotion. However, miR-137 cKO mice displayed a reduction in time spent in the center (Figure 1F), and fewer center entries (Figure 1G) compared to the control group. These results suggest that loss of miR-137 in the nervous system lead to anxiety-like behavior in mice.

### Loss of miR-137 Results in Dendritic and Synaptic Overgrowth

**Figure 2:**
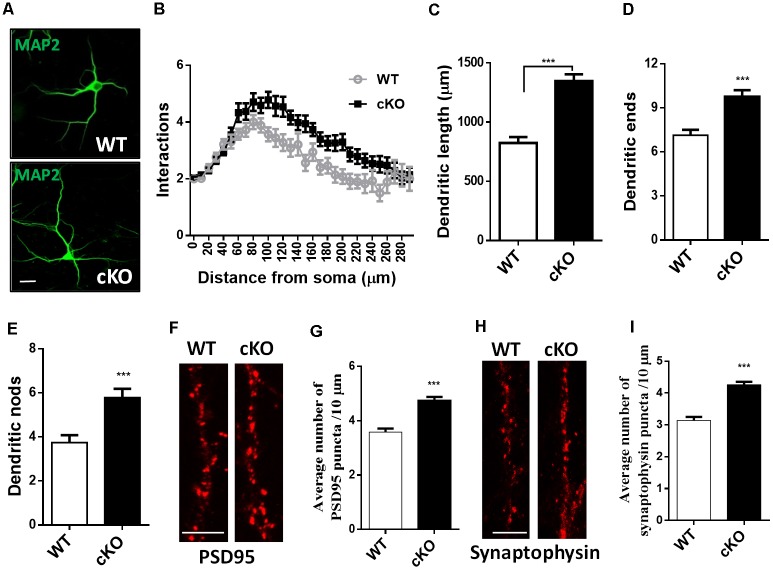
Hippocampal neurons were fixed at DIV7 and immunostained against MAP-2 for neurites analysis, or fixed at DIV21 and immunostained against PSD95 or synaptophysin for synapse analysis. **(A)** Representative images of MAP-2 immunostaining on hippocampal neurons at DIV7. Scale bar, 20 μm. **(B)** Sholl analysis of hippocampal neurons demonstrated that loss of miR-137 significantly increased the number of intersection at 60–240 μm from the soma compared to non-treated neurons (*n* = 46–52 neurons from three to four mice, ***p < 0.001, two-way ANOVA). miR-137 cKO neurons showed increased dendritic length **(C)**, dendritic ends **(D)** and number of nodes (branch points, **E**; *n* = 46–52 neurons from three to four mice, ***p < 0.001, two way ANOVA). **(F)** Cultured hippocampal neurons were fixed and stained for PSD-95. **(G)** Quantification of average PSD95 puncta intensity demonstrated that miR-137 cKO neurons had increased number of PSD95 puncta (*n* = 43 neurons per genotype; ***p < 0.001). Scale bar, 5 μm. **(H)** Cultured hippocampal neurons were fixed and stained for synaptophysin. **(I)** The average number of synaptophysin puncta was also increased in miR-137 cKO neurons (*n* = 40 neurons per genotype; ***p < 0.001). Scale bar, 5 μm.

To determine whether the loss of miR-137 influences dendritic and synaptic growth, we isolated neurons from the hippocampi of E17.5 miR-137 cKO or WT mouse embryos and cultured them in serum-free medium to limit astrocyte proliferation. Hippocampal neurons were fixed at DIV7 and immunostained against MAP-2 for neurites analysis (Figure 2A), or fixed at DIV21 and immunostained against PSD95 or synaptophysin for synapse analysis (Figures 2F,H). Sholl analysis indicated that miR-137 cKO hippocampal neurons exhibited significantly increased dendritic complexity compared with WT neurons (Figure 2B). Moreover, miR-137 cKO hippocampal neurons exhibited significantly dendritic length (Figure 2C), number of dendritic ends (Figure 2D), and number of dendritic nodes (Figure 2E), indicating that miR-137 loss-of-function resulted in dendritic overgrowth.

Immunocytochemistry and quantification of PSD95 puncta demonstrated that miR-137 cKO hippocampal neurons had increased number of PSD95 puncta (Figure 2G). In addition, the number of synaptophysin puncta was also substantially increased in miR-137 cKO neurons compared to WT neurons (Figure 2I). Altogether, these findings suggest that synapse overgrowth upon loss of miR-137.

### MiR-137 cKO Mice Exhibit Altered Synaptic Transmission and Plasticity

**Figure 3:**
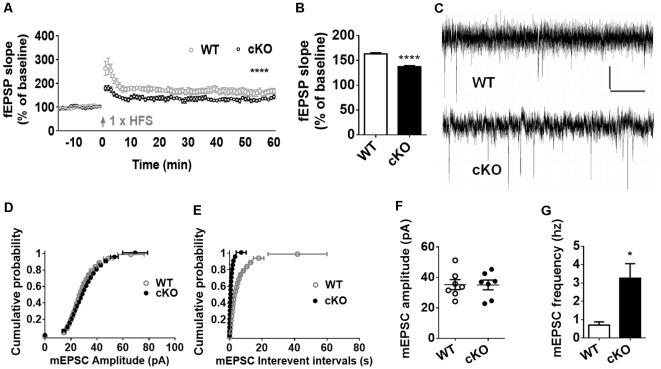
MiR-137 cKO mice exhibit alterations in synaptic plasticity. **(A)** SC-CA1 synapses in the miR-137 cKO hippocampus showed suppressed long-term potentiation (LTP) induced by high-frequency stimulation (HFS), compared to WT littermate controls (*n* = 5 slices from four mice for WT and 6, 4 for cKO, ****p < 0.0001, two way ANOVA). Arrows indicate application of HFS. **(B)** LTP magnitude presented as mean fEPSP slope of the last 5 min (55–60 min) of recording. **(C)** Traces showing mEPSCs recorded from primary cultured hippocampal neurons from WT (Anacker and Hen, 2017) and miR-137 cKO (bottom) mice, scale bar: 10 pA, 1 s. **(D,E)** Cumulative probability, and summarized mean values **(F,G)** of mEPSCs amplitude **(D,F)** and frequency **(E,G)** recorded from primary cultured hippocampal neurons from miR-137 cKO mice and their WT littermates (*n* = 7, *p < 0.05, student’s *t*-test).

Altered synaptic plasticity has been implicated as a mechanism that may contribute to brain dysfunction, such as dementia, neuropathic pain, depression and anxiety (Lüscher and Malenka, 2012). With observed above anxiety-like behavior and dendritic and synaptic overgrowth, we further investigated the effect of miR-137 loss-of-function in neuronal plasticity. We recorded extracellular field EPSP to examine whether the deletion of miR-137 disrupts normal synaptic transmission at hippocampal Schaffer collateral CA3-CA1 synapses. LTP induction by *in vitro* HFS led to an overall increase in synaptic strength in both miR-137 cKO and WT groups, but there was a significant reduction in the synaptic potentiation in miR-137 cKO mice compared with their WT littermates (Figure 3A). The mean fEPSP slope at 55–60 min after LTP induction was significantly attenuated in miR-137 cKO mice (Figure 3B).

Subsequently, we measured the frequency and amplitude of mEPSCs of hippocampal neurons in the presence of 1 μM TTX and 100 μM PTX (Figures 3C–G). Compared to the control group, the mean amplitude of mEPSCs was unchanged in miR-137 cKO mice (Figure 3F), as reflected in the similar cumulative probability distribution of mEPSC amplitudes to WT controls (Figure 3D). The cumulative frequency of interevent intervals was shifted leftward, indicating smaller interevent intervals in miR-137 cKO mice (Figure 3E). In addition, the mean frequency of mEPSCs was significantly increased in miR137 cKO neurons (Figure 3G). Therefore, loss of miR-137 resulted in impaired synaptic transmission and plasticity in the hippocampus.

### *Ezh2* Is Upregulated in the Forebrain of miR-137 cKO Mice

**Figure 4:**
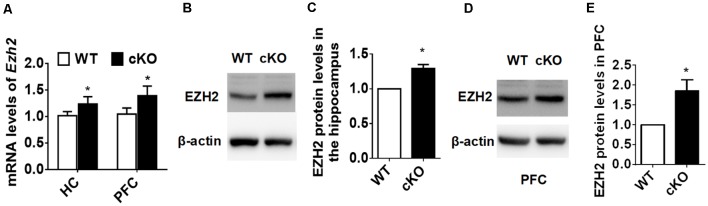
EZH2 is upregulated in the forebrain of miR-137 cKO mice. **(A)** qPCR analysis revealed that *Ezh2* mRNA levels were significantly higher in the hippocampus and PFC of miR-137 cKO mice than those of the WT littermates (*n* = 6, *p < 0.05, student’s *t*-test). **(B–E)** Western blot analysis revealed that EZH2 protein levels were also increased in the hippocampus **(B,C)** and PFC **(D,E)** of miR-137 cKO mice (*n* = 3, *p < 0.05, student’s *t*-test).

To explore the molecular mechanisms through which miR-137 exerts its function in mood disorders, we used the TargetScan algorithm (Agarwal et al., 2015) and the miRTarBase database (Chou et al., 2018), and identified 125 genes experimentally validated as having a miR-137 target site. We then searched MEDLINE to select among these genes those relevant to synaptic plasticity and neurodevelopment, especially those with a known downregulated expression in patients with mood disorders. We used each one of the 125 gene names together with the terms “anxiety OR depression OR synaptic plasticity OR neurodevelopment.” Out of several potential targets, we screened those that would match with our observation of synaptic overgrowth and mental dysfunction in miR-137 deletion mice. EZH2 fulfilled these criteria. Using qPCR, we confirmed that *Ezh2* mRNA expression levels were significantly higher in the hippocampus and PFC of adult miR-137 cKO mice compared to WT littermates (Figure 4A). EZH2 protein levels in both the hippocampus (Figures 4B,C) and PFC (Figures 4D,E) were significantly increased as well.

### Knockdown of *Ezh2* Rescues the Deficits Associated With the Loss of miR-137

**Figure 5:**
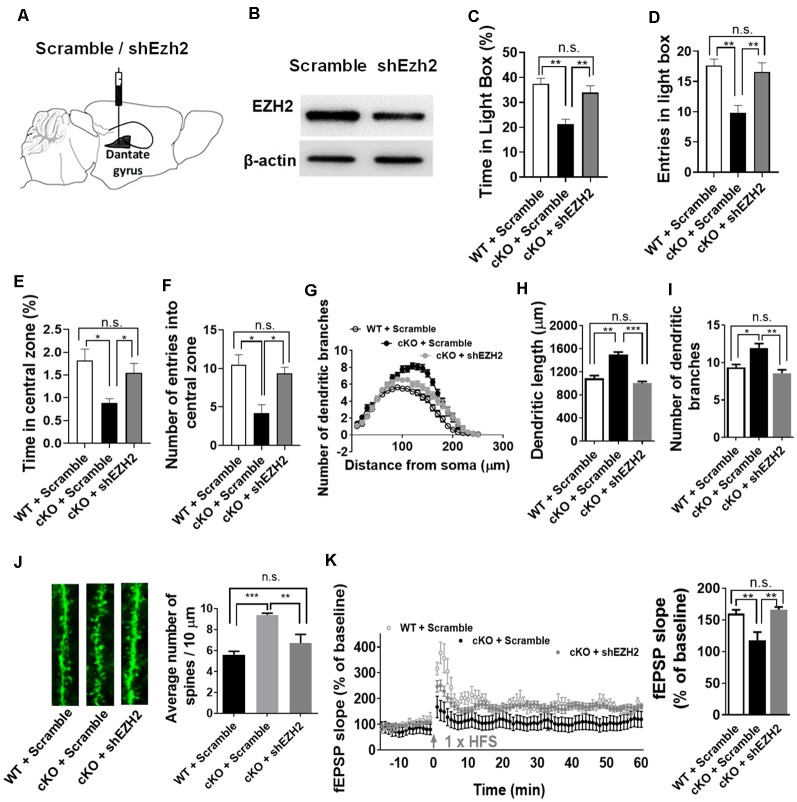
Knockdown of *Ezh2* rescues the deficits associated with the loss of miR-137. **(A)** Diagram showing that lentivirus encoding shRNA targeting *Ezh2* (shEzh2) or negative control (Scramble) were injected into the dentate gyrus (DG). **(B)** The shEzh2 lentivirus could efficiently knock down the expression of EZH2 protein in the hippocampus. **(C,D)** In the light/dark box test, shEzh2 mice demonstrated improved time **(C)** and entries **(D)** in light box (cKO + shEZH2 vs. cKO + Scramble: *n* = 12–14 mice per group, **p < 0.01). **(E,F)** In the open field test, shEzh2 reduced the anxious levels in miR-137 cKO mice, as indicated by significantly increased time **(E)** spent in the central zone (cKO + shEZH2 vs. cKO + Scramble: *n* = 12–14 mice per group, *p < 0.05) and more entries **(F)** in it (cKO + shEZH2 vs. cKO + Scramble: *n* = 12–14 mice per group, *p < 0.05). **(G)** sh-EZH2 dramatically reduced dendritic complexity in miR-137 deletion neurons compared with controls, as determined by Sholl analysis (*n* = 35–42 neurons per group, ***p < 0.01, ***p < 0.001). **(H)** sh-EZH2 significantly reduced dendritic length (*n* = 35–42 neurons per group, **p < 0.01, ***p < 0.001). **(I)** sh-EZH2 had a significant effect on the number of dendritic branches (*n* = 35–42 neurons per group, *p < 0.05, **p < 0.01). **(J)** Knockdown of EZH2 in miR-137 cKO neurons reduced the spine density to a degree similar to that seen in WT group (*n* = 38–40 neurons per group, **p < 0.01, ***p < 0.001). Left: representative images of dendritic spines. Right: statistical analyses of spine density. **(K)** sh-EZH2 rescued the impaired LTP in miR-137 cKO mice. Left: pooled time-course data of LTP from all recordings made from miR-137 cKO + scramble, miR-137 cKO + shEZH2, and miR-137 WT + scramble mice. Right: average LTP amplitude measured 55–60 min post-induction (*n* = 5–6 slices from four mice per group, **p < 0.01). n.s., non-significant.

Elevated EZH2 expression has been shown in individuals with severe anxiety (Murphy et al., 2015). Given the substantial increase of EZH2 protein in the forebrain upon the loss of miR-137, we thus reasoned that knockdown of *Ezh2* might ameliorate the anxious behaviors of miR-137 cKO mice. To test this, we injected lenti-shEZH2 virus in the hippocampi of adult mir-137 cKO and WT mice (Figure 5A) and assessed the impact of EZH2 inhibition on the abnormal mental phenotypes associated with the loss of miR-137.

Our western blot assays confirmed the high knockdown efficiency of lenti-shEZH2 in the hippocampi of miR-137 cKO mice (Figure 5B). In the light-dark box text, sh-EZH2 drastically ameliorated the anxiety-like behavior in miR-137 cKO mice, as reflected in increased time (Figure 5C) and entries (Figure 5D) in the light compartment. In the open field test, shEzh2 improved the time spent in the central zone (Figure 5E) and the number of entries in it (Figure 5F).

We also found that knockdown of EZH2 could significantly reduce the dendritic complexity (Figure 5G), dendritic length (Figure 5H), number of dendritic branches (Figure 5I), and spine density in miR-137 cKO mice (Figure 5J). Meanwhile, EZH2 inhibition could also rescue the impaired LTP (Figure 5K) and spatial learning and memory (Supplementary Figures S1D,E) in miR-137 cKO mice.

## Discussion

Anxiety and depression are major public health concerns, but the underlying molecular and cellular mechanisms remain elusive and current treatments aim mostly at alleviating the symptoms rather than curing the disorder. MicroRNAs are believed to be reliable biomarkers in the diagnosis and treatment of mood disorders (Li et al., 2013; Fan et al., 2014; Wan et al., 2015; Wang et al., 2015; Lopez et al., 2017; Roy et al., 2017; Tavakolizadeh et al., 2018). Emerging pre-clinical and clinical evidence suggest that dysregulation of microRNAs may be critical for the development of depression and suicidal behavior (Dwivedi, 2018). For example, miR135a levels are deduced in the blood and brain of depressed human patients (Issler et al., 2014). Similarly, genetically modified mice which express higher or lower levels of miR135 display depression- and anxiety-like behaviors (Issler et al., 2014). A microRNA-screening study has reported that miR-137 levels are substantially down-regulated by 25% in the postmortem PFC of depressed patients with suicidal behavior (Smalheiser et al., 2012). The expression level of miR-137 is significantly lower in the brain in post-stroke depression rats, and delivery of miR-137 into the brain improves their behavioral performance by suppressing the expression of Grin2A (Zhao et al., 2013). In addition, several reports have provided strong association between CACNA1C, a potential target gene of miR-137, with the risk of major depression (Casamassima et al., 2010; Green et al., 2010; Shi et al., 2011). Here our results suggest that the loss of miR-137 in the forebrain potentially causes anxiety by highly elevated EZH2.

*MiR-137* is highly enriched in brain (Sun et al., 2011), and dramatically down-regulated in neuroblastoma (Althoff et al., 2013) and glioblastoma multiforme (Silber et al., 2008). MiR-137 plays important roles in development as well as in neurodevelopmental disorders such as schizophrenia (Agarwal et al., 2015). *Mir137* mutant mice display embryonic lethality that occurs after implantation (~E4.5) but before E11.5 (Crowley et al., 2015). In our previous study, we specifically deleted *Mir-137* in the germline or nervous system of the mice and found that mice completely lacking *Mir137* have problems with development and die soon after birth (Cheng et al., 2018). Our present study demonstrated that mice with specific deletion of *Mir137* in the forebrain could survive to reproduce, suggesting that miR-137 has multiple roles in different brain areas and/or organs.

*Mir-137* is highly conserved and plays a critical role in neurogenesis and neuronal maturation (Smrt et al., 2010; Szulwach et al., 2010; Sun et al., 2011; Hollins et al., 2014; Mahmoudi and Cairns, 2017). Lentivirus transduction studies showed that overexpression of miR-137 inhibits dendritic morphogenesis and spine development, while downregulation of miR-137 had opposite effects (Smrt et al., 2010). MiR-137 gain of function by lentivirus transduction resulted in a notably vesicle-sparse region in the mossy fiber presynaptic terminals, impaired induction of mossy fiber LTP and deficits in hippocampus-dependent learning and memory (Siegert et al., 2015). In a recent study, He and colleagues confirmed that over-expression of miR-137 significantly changes the synaptic transmission, but without any selective synaptic vesicle docking defects (He et al., 2018).

Systematic review and meta-analysis support the idea that adult patients with autism spectrum disorder are thought to be at disproportionate risk of developing anxiety and depression (Hollocks et al., 2019). We previously found that mice with one copy of *Mir137* disrupted in the brain display autism-like behavior, dendritic and synaptic overgrowth, and impaired learning and social behavior (Cheng et al., 2018). In the present study, we confirmed that miR-137 loss-of-function in the forebrain led to dendritic and synaptic overgrowth, altered synaptic plasticity, and intellectual disability. Moreover, mice with deletion of *MIR-137* in the forebrain displayed anxiety-like behavior, as indicated by a significant decrease in both the time spent and the number of entries in the light chamber in the light-dark preference test, and by reductions in the time spent and the number of entries in the center in the open field test. Therefore, our *in vivo* studies supported that miR-137 loss-of-function is closely associated not only with autism, but also with anxiety.

EZH2, a histone-lysine N-methyltransferase enzyme that participates in histone methylation (Vire et al., 2006), is an epigenetic regulator in regulating the proliferation and differentiation of neural stem/progenitor cells (NSPCs; Pereira et al., 2010; Hwang et al., 2014; Zhang et al., 2014; Liu et al., 2017). *Ezh2* has been experimentally validated as a direct target of miR-137 in mouse neural stem cells (Szulwach et al., 2010), and also in human glioblastoma cells (Sun et al., 2015) and neuroblastoma cells (Ren et al., 2015) by luciferase reporter gene analysis. Significantly higher levels of global DNA methylation have been discovered in anxious patients compared with controls (Murphy et al., 2015). Interestingly, overexpress of miR-101a-3p in the amygdala of high-novelty-responding (HR) rats increased their anxiety-like behavior at least partially mediated *via* downregulation of EZH2 (Cohen et al., 2017). Here we observed a higher expression level of EZH2 in the forebrain of miR-137 cKO mice that displayed anxiety-like behavior. Importantly, knockdown of EZH2 could ameliorate the anxious phenotypes in miR-137 cKO mice. Given that EZH2 is a well-characterized transcription repressor that is highly expressed in NSPCs but decreased upon neuronal differentiation (Liu et al., 2017), an important question that remains unanswered is how the upregulation of EZH2 contribute to anxiety. Future studies to elucidate these mechanisms might be very helpful to discover novel molecule targets for treating mood disorders.

In summary, we show here that the loss of miR-137 in the forebrain results in cellular, electrophysiological, and behavioral alterations that might be responsible for anxiety, and we identify EZH2 as a potential therapeutic target for anxious phenotypes associated with the loss-of-function of miR-137.

## Data Availability Statement

All datasets for this study are included in the article/Supplementary Material.

## Ethics Statement

The animal study was reviewed and approved by Institute of Zoology, Chinese Academy of Sciences.

## Author Contributions

Z-QT, C-ML, H-LY, and P-PL: conception and design, collection and assembly of data, data analysis and interpretation, manuscript writing, and final approval of manuscript. X-WS, Z-MW, T-WM, CL, Y-YW, X-CH, and H-ZD: collection and assembly of data, and final approval of manuscript.

## Conflict of Interest

The authors declare that the research was conducted in the absence of any commercial or financial relationships that could be construed as a potential conflict of interest.

## Supplementary Material

FIGURE S1 Loss of miR-137 leads to the spatial learning and memory deficits, and shEzh2 restores the spatial learning performance of miR-137 cKO mice. **(A)** During the training phase, in which 4 trials were conducted per day for 5 successive days, miR-137 cKO mice exhibited a significant delay to diminish the latency to locate the platform (*n* = 14–17 mice per group, **p* < 0.05, student’s *t*-test). **(B)** In probe trials on Day 6, miR-137 cKO mice displayed a significantly longer latency to locate the platform (*n* = 14–17 mice per group, **p* < 0.05, student’s *t*-test), but **(C)** fewer target crossings (*n* = 14–17 mice per group, **p* < 0.05, student’s *t*-test). **(D)** shEzh2 restored the spatial learning performance of miR-137 cKO mice in Morris water maze test (*n* = 14–17 mice per group, **p* < 0.05, Two-way ANOVA). **(E)** shEzh2 treated mice had a shorter latency to find the previous hidden platform in the probe test of Morris water maze test compared to scramble controls (*n* = 13–18 mice per group, **p* < 0.05, ***p* < 0.01, Two-way ANOVA).
