# Supplementary figures and images for: MiR-137 Deficiency Causes Anxiety-Like Behaviors in Mice
Source: Front Mol Neurosci. 2019 Oct 30;12:260. doi: 10.3389/fnmol.2019.00260 (PMC6831983; doi:10.3389/fnmol.2019.00260)

Supplementary Figure 1

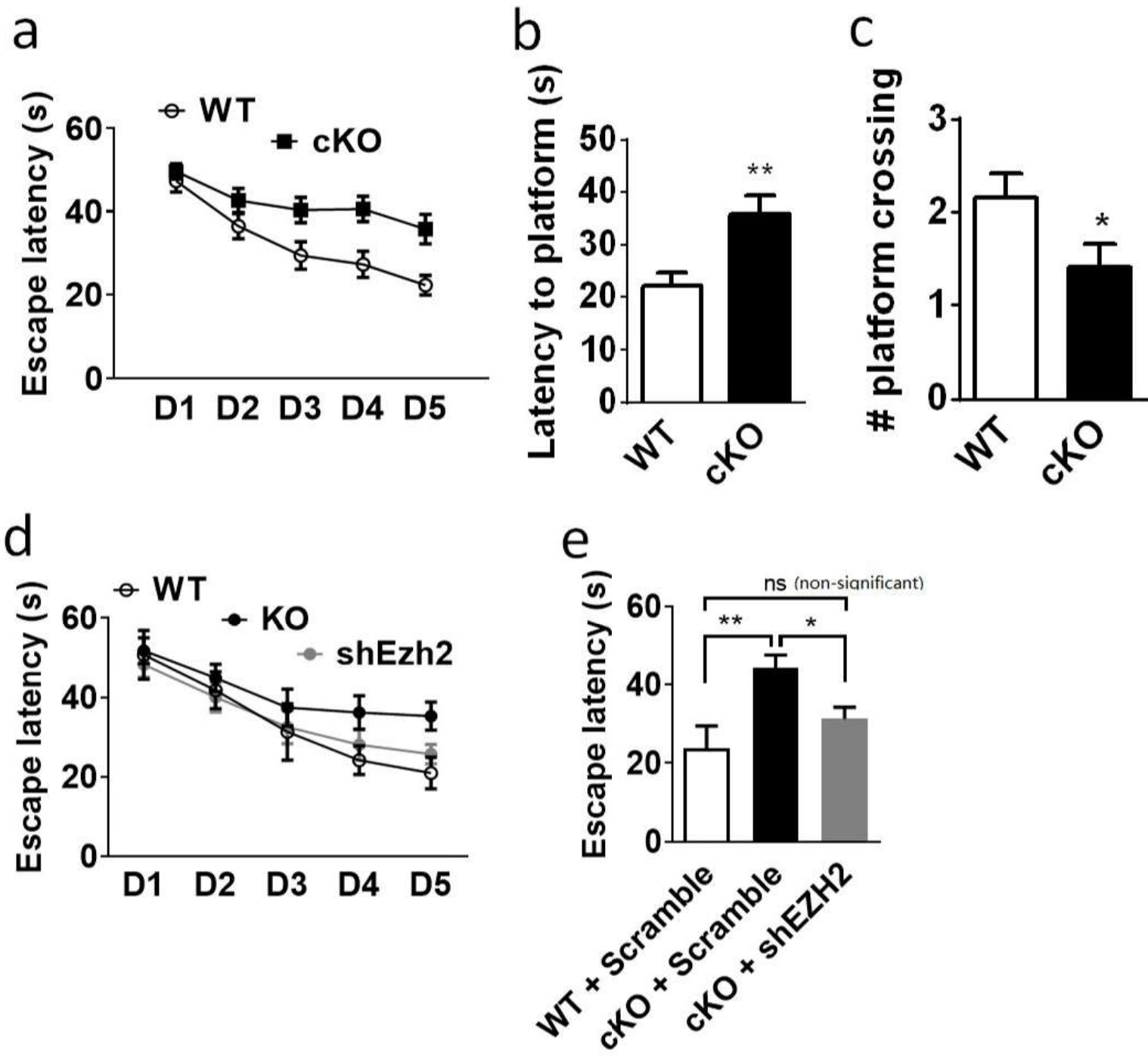

Supplement: FIGURE S1 — Loss of miR-137 leads to the spatial learning and memory deficits, and shEzh2 restores the spatial learning performance of miR-137 cKO mice. (A) During the training phase, in which 4 trials were conducted per day for 5 successive days, miR-137 cKO mice exhibited a significant delay to diminish the latency to locate the platform (n = 14–17 mice per group, *p < 0.05, student’s t-test). (B) In probe trials on Day 6, miR-137 cKO mice displayed a significantly longer latency to locate the platform (n = 14–17 mice per group, *p < 0.05, student’s t-test), but (C) fewer target crossings (n = 14–17 mice per group, *p < 0.05, student’s t-test). (D) shEzh2 restored the spatial learning performance of miR-137 cKO mice in Morris water maze test (n = 14–17 mice per group, *p < 0.05, Two-way ANOVA). (E) shEzh2 treated mice had a shorter latency to find the previous hidden platform in the probe test of Morris water maze test compared to scramble controls (n = 13–18 mice per group, *p < 0.05, **p < 0.01, Two-way ANOVA). [file Image_1.pdf]
